# Optimization of Roasting Process and Thermal Parameter Adaptability for Guisha Limonite Pelletizing

**DOI:** 10.3390/ma19122444

**Published:** 2026-06-08

**Authors:** Yanjing Bai, Xiaolei Zhou, Xiaotian Ma

**Affiliations:** Faculty of Metallurgical and Energy Engineering, Kunming University of Science and Technology, Kunming 650093, China; 202310201521@stu.kust.edu.cn (Y.B.);

**Keywords:** limonite, thermal decrepitation, compressive strength, preheating temperature

## Abstract

**Highlights:**

- Guisha limonite pelletizing was optimized by considering bentonite dosage, pellet size, and thermal regime.
- The optimal thermal regime was preheating at 700 °C, roasting at 1250 °C, and slow furnace cooling.
- Bentonite dosages of 1.2 wt% and 1.4 wt% were proposed for strength-prioritized and anti-decrepitation-prioritized production, respectively.

**Abstract:**

Driven by the urgent demand of the steel industry for utilizing low-grade, high-crystal-water iron ores, this study focuses on the thermal decrepitation problem in Guisha limonite pellet preparation caused by goethite dehydroxylation. Different from previous studies that mainly focused on single factors or single performance indicators, this work establishes a multi-factor experimental framework that simultaneously considers bentonite dosage, preheating temperature, and pellet size. This framework enables the strength–decrepitation trade-off of Guisha limonite pellets to be evaluated quantitatively rather than empirically. This work systematically investigated bentonite addition (0.8–1.6 wt%), preheating temperature (600–800 °C), and pellet diameter (9–13 mm). These factors were evaluated in terms of thermal cracking mass ratio and compressive strength. Their interactive effects on thermal cracking behavior and mechanical properties were quantitatively revealed. A target-oriented dual-window process control strategy was then proposed. The results show that thermal cracking intensifies with increasing preheating temperature and decreases with increasing bentonite content; compressive strength peaks at 1.2 wt% bentonite (approx. 1456 N). On this basis, a Min–Max normalization and weighted scoring method was adopted. A quantitative decision-making model was established for strength-prioritized and safety-prioritized objectives. The model identified two optimal process control windows at 1.2 wt% and 1.4 wt% bentonite. An optimized thermal regime—preheating at 700 °C, roasting at 1250 °C, and slow furnace cooling—was established. This regime provides directly referable process parameters. It also offers a decision-making framework for pellet production of similar ores.

## 1. Introduction

Owing to the sustained expansion of the global iron and steel sector, the demand for iron ore resources has persisted at elevated levels for an extended period [1]. Following decades of intensive mining activities, the average grade of accessible iron ores has undergone a progressive decline, thereby necessitating enhanced efforts toward the exploitation of ores that are traditionally more challenging to process. In this context, iron oxide resources that contain structurally bound crystal water and are geographically widespread have begun to attract increasing research and industrial interest [2,3,4,5,6,7]. The judicious development and systematic utilization of these vast reserves of low-grade, mineralogically complex iron ores are of considerable strategic importance. Such efforts are vital for safeguarding the security of raw material supply chains that underpin China’s steel industry and for diminishing reliance on imported, high-grade ore concentrates. Facilitated by ongoing breakthroughs in metallurgical technologies, limonite is steadily transitioning from the status of a “marginal resource” to that of a significant alternative iron-bearing feedstock for industrial operations. Compared with the conventional sintering route, pelletizing technology offers several distinct benefits. These include reduced energy intensity [8,9,10], improved capacity for emission reduction [11,12,13], and superior adaptability to lean ore feeds. Consequently, pelletizing is increasingly regarded as a more economically attractive processing option [14,15,16]. The pelletizing sequence typically entails grinding the raw ore into a fine powder and sieving such that particles passing a 200-mesh screen constitute in excess of 85 percent of the total mass. The powder is then blended with a suitable binder [17,18,19] and shaped into green pellets within a disc pelletizer [20]. The initial properties of green pellets include handling strength and dimensional uniformity. These properties depend on the particle size distribution of the powder, the binder type and dosage, the initial moisture level, and the final pellet dimensions [21].

The Guisha limonite investigated in this study possesses notably complex mineralogical attributes [22]. Analysis via X-ray diffraction (XRD) demonstrates that the principal iron-bearing phases are goethite (α-FeO(OH)) and hematite (α-Fe_2_O_3_). Goethite accommodates a considerable amount of structural water within its crystalline framework, and the loss on ignition (LOI) of the raw ore is documented to be as high as 14.82% [23,24]. Further microstructural examination using scanning electron microscopy (SEM) discloses that the surfaces of the ore particles exhibit a coral-like, loosely porous morphology [25,26,27,28,29]. This distinctive microstructural signature exerts a dual influence. On one hand, the pronounced hydrophilicity of the ore promotes facile inter-particle adhesion, which translates into excellent drop strength and balling efficiency for the green pellets. On the other hand, the combination of a high LOI value and an intrinsically porous architecture engenders a significant vulnerability to thermal decrepitation. During rapid heating, goethite dehydroxylation proceeds vigorously. The generated vapor pressure can readily exceed the mechanical accommodation capacity of the pellet structure and induce rupture. The released water vapor faces strong resistance when escaping through narrow, tortuous pore channels. This leads to a precipitous accumulation of internal pore pressure. Once this pressure exceeds the tensile strength of the solid skeleton, macroscopic fragmentation or even explosive pulverization occurs [30,31,32]. This phenomenon is widely known as “thermal decrepitation.” The fine particulate debris produced by decrepitation can block void spaces in the blast furnace burden column. This severely impairs gas permeability and threatens stable and safe blast furnace operation [33,34].

The compressive strength ultimately attained by finished pellets is closely related to the densification mechanisms that govern the behavior of Guisha limonite. This densification process can be divided into three stages. First, Fe_2_O_3_ crystallites nucleate and initially develop. Second, these grains expand and coarsen. Third, intergranular connectivity is established, forming a coherent dense body through solid-state consolidation [35]. Experimental evidence shows clear temperature-dependent crystallization behavior. Primary crystals of Fe_2_O_3_ begin to form at approximately 1150–1200 °C. Mature crystalline structures develop between 1220 and 1250 °C. An interconnected crystalline network is achieved at temperatures above 1280 °C [36,37,38]. Therefore, two core and interdependent criteria determine the viability of limonite pelletization. Thermal decrepitation must be suppressed throughout the roasting cycle, and the final indurated pellets must retain adequate compressive strength. The optimization of process parameters, accordingly, must navigate and reconcile a delicate equilibrium between these competing demands.

Wen et al. reported that straw powder can substantially elevate the decrepitation temperature of pellets fabricated from ultrafine iron ore concentrates. This improvement, however, is accompanied by a pronounced decrease in cold compressive strength [39]. In a separate investigation on the roasting characteristics of Yunnan limonite pellets, Chen et al. noted that changing the bentonite addition level could align compressive strength with the general requirements of blast furnace operation [40]. Despite these valuable contributions, the existing literature still lacks a systematic understanding of binder ratio, thermal regime, and pellet size acting together. Their combined effects on microstructural consolidation and macroscopic performance also remain insufficiently clarified. Accordingly, this study takes Guisha limonite as the research object. It innovatively constructs a multi-factor synergistic experimental system that includes bentonite ratio, thermal regime, and pellet size. The study aims to (1) quantitatively reveal the interactive influence of the three factors on thermal cracking behavior and compressive strength; (2) introduce a normalized weighted scoring method to elevate the process trade-off from qualitative judgment to quantitative decision-making; and (3) formulate an optimized combination of thermal parameters that can directly guide industrial production. This work fills the gap in multi-factor synergy and process decision-making in existing studies. It also provides a scientific and practical technical reference for the large-scale pelletizing of similar high-crystal-water iron ores.

## 2. Materials and Methods

### 2.1. Experimental Materials

#### 2.1.1. Guisha Limonite

**Figure 1:**
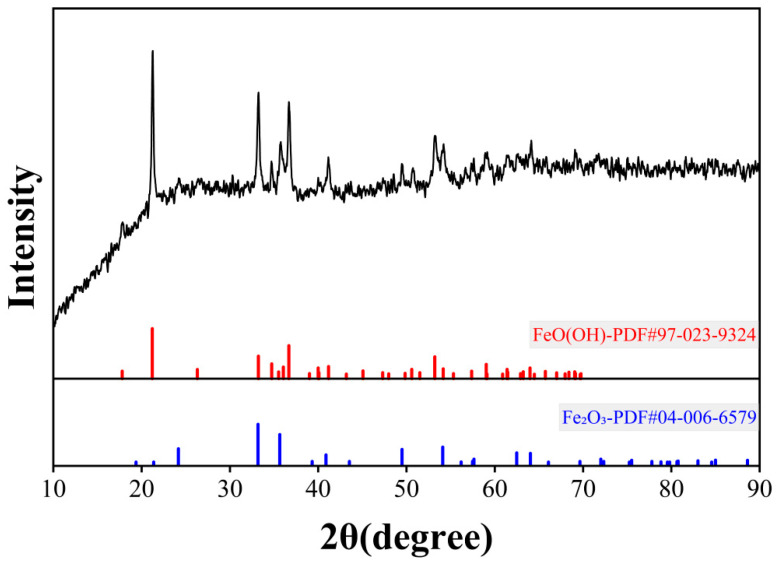
XRD pattern of Guisha limonite.

The Guisha limonite used in the experiments was sourced from an iron ore processing plant affiliated with Kunming Iron and Steel Group in Kunming, Yunnan Province, China. Its main chemical composition is presented in Table 1. The ore has a total iron (TFe) grade of 54.67%, with the principal gangue components being SiO_2_ and MnO, and a loss on ignition (LOI) as high as 14.82%. X-ray diffraction (XRD) analysis results are shown in Figure 1. The characteristic peaks in the pattern confirm that the main iron-bearing phase is goethite (α-FeO(OH)), accompanied by a minor amount of hematite (α-Fe_2_O_3_). Goethite is an iron oxide hydroxide containing structurally bound crystal water, and its thermal decomposition behavior is the root cause of thermal decrepitation during pellet roasting.

**Figure 2:**
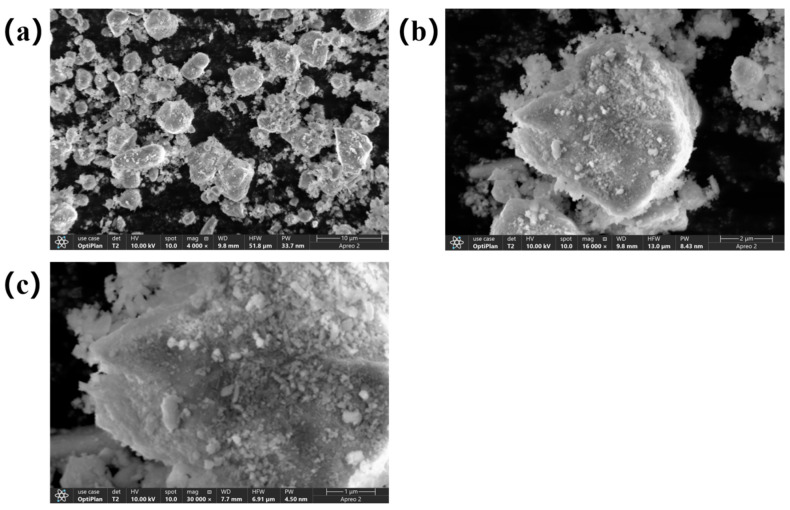
SEM images of Guisha limonite powder at different magnifications: (**a**) 4000×; (**b**) 16,000×; (**c**) 30,000×.

Scanning electron microscopy (SEM) (Thermo Fisher Scientific, Waltham, MA, USA) was employed to characterize the microstructure of the ore powder, with the results presented in Figure 2. At low magnification (Figure 2a), the particle surfaces appear rough and angular; at high magnifications (Figure 2b,c), the particle surfaces exhibit dense nanoscale micropores and a loose, friable structure. This porous morphology imparts two characteristics to the ore: on one hand, the strong hydrophilicity facilitates particle agglomeration into pellets, which is beneficial for improving the drop strength and balling efficiency of green pellets; on the other hand, the combination of a high LOI and the porous structure superimposes a severe risk of thermal decrepitation—during rapid heating, moisture vaporizes violently while the diffusion channels are restricted, leading to a sharp increase in internal vapor pressure.

#### 2.1.2. Bentonite

The binder selected was a high-quality sodium-based bentonite supplied by Kunming Iron and Steel Group (Kunming, China), whose physicochemical performance indices are listed in Table 2. The montmorillonite content is 85.97%, and the water absorption and swelling capacity are at relatively high levels, indicating that the bentonite possesses good binding properties capable of meeting the requirements of pellet preparation.

### 2.2. Experimental Equipment and Measuring Instruments

Pellet preparation was carried out using a 6GU-15K disc pelletizer (Zhengzhou Ruichang Machinery Equipment Co., Ltd., Zhengzhou, China). Ore powder grinding was performed using a QHQM-100 ball mill (Hunan Qinghe Heavy Industry Machinery Co., Ltd., Changsha, China). Preheating experiments were conducted in an STGL-60-17 resistance furnace (Henan Sante Furnace Technology Co., Ltd., Luoyang, China), and roasting experiments were performed in an STM-12-14 muffle furnace (Henan Sante Furnace Technology Co., Ltd., Luoyang, China).

Phase analysis was performed using a Rigaku MiniFlex600 X-ray diffractometer (Rigaku Corporation, Tokyo, Japan). Microstructural observation was conducted using an Apreo 2S field-emission scanning electron microscope (Thermo Fisher Scientific, Waltham, MA, USA). The compressive strength of finished pellets was tested using an SKE-10KN digital display pressure tester (Hunan Zhenhua Analysis Instrument Co., Ltd., Xiangtan, China).

### 2.3. Experimental Methods

**Figure 3:**
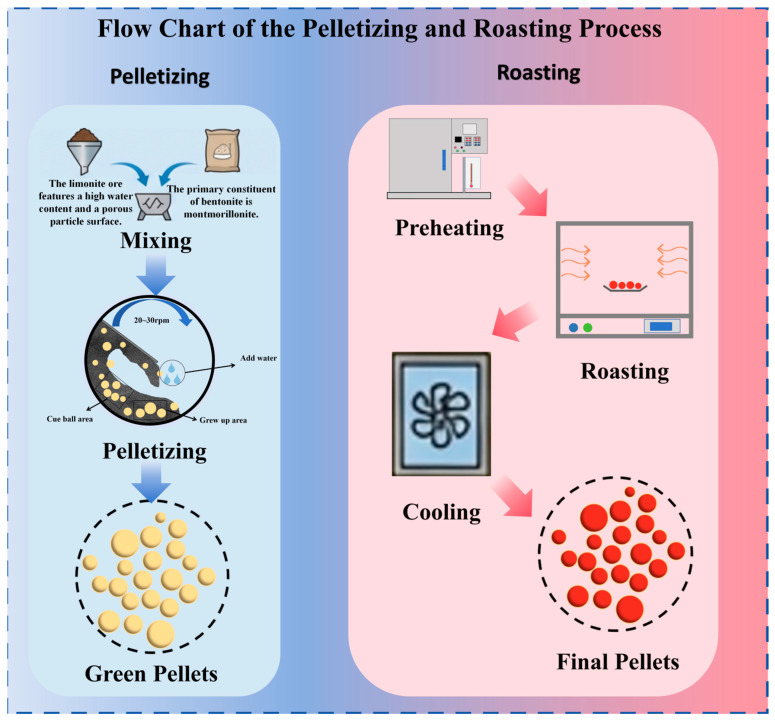
Flowchart of the pelletizing and roasting processes.

The overall experimental flow is illustrated in Figure 3. The Guisha limonite was ground to a fineness with over 85% passing a 200-mesh screen, mixed with bentonite according to the predetermined proportions, homogenized, and then moistened with water for pelletizing. The resulting green pellets were screened, and pellets with diameters in the range of 9–13 mm were selected as experimental samples.

To systematically investigate the influence of various factors on pellet performance, the following three series of experiments were designed:

#### 2.3.1. Experiment 1: Thermal Decrepitation Behavior

This set of experiments aimed to investigate the synergistic effects of bentonite proportion and rapid heating on the thermal decrepitation behavior of pellets.

Green pellets with bentonite additions of 0.8 wt%, 1.0 wt%, 1.2 wt%, 1.4 wt%, and 1.6 wt% were selected. They were then subjected to heating decrepitation experiments in an STGL-60-17 resistance furnace. The experimental parameters were set as follows: an air flow rate of 10 m^3^/h, an air velocity of 1.5 m/s, and a fixed heating time of 30 min. The green pellets were heated from room temperature to preset target temperatures of 600 °C, 650 °C, 700 °C, 750 °C, and 800 °C.

The design of a fixed heating time was intended to simulate the actual thermal shock experienced by green pellets when directly entering preheating sections at different temperatures. Under these conditions, a higher target temperature corresponds to a steeper average heating rate, and the two are positively correlated.

For each condition, 60–80 pellets were tested as one batch to reduce random error caused by individual pellet variability. The green pellets were dried in an oven at 200 °C for 2 h. This step removed free moisture. The total mass of the pellets after drying was then weighed and recorded. The pellets were placed into the resistance furnace and subjected to the thermal shock experiment according to the set heating regime. After the experiment, pellet fragments and powder caused by obvious rupture, spalling, or pulverization due to thermal decrepitation were collected and weighed.

The thermal cracking mass ratio (*R_m_*) was used as the indicator to characterize the degree of thermal cracking, calculated as shown in Equation (1):

(1) $$R_{m}=\frac{m_{d}}{m_{0}}\times 100\%$$

Here, $R_{m}$ is the thermal cracking mass ratio (%). $m_{d}$ is the mass of pellets that have undergone obvious rupture after thermal shock (g). $m_{0}$ is the total mass of the pellets after moisture removal (g). A larger $R_{m}$ value indicates a more severe degree of thermal cracking.

#### 2.3.2. Experiment 2: Consolidation Strength

This set of experiments aimed to investigate the comprehensive effect of bentonite proportion and pellet diameter on the compressive strength of finished pellets.

The fixed process parameters were a preheating temperature of 700 °C, a preheating time of 10 min, a roasting temperature of 1250 °C, and a roasting time of 25 min. Under these conditions, green pellets with different bentonite proportions (0.8 wt–1.6 wt%) and different diameters (9–13 mm) were placed in a muffle furnace for roasting. After roasting, the finished pellets were cooled to room temperature in the furnace.

Compressive strength testing method: a pellet was placed between the upper and lower platens of the digital display pressure tester and loaded radially at a constant rate until the pellet fractured. The maximum load value at the moment of rupture was recorded. For each set of experimental conditions, 10 finished pellets were tested, and the results are expressed as mean ± standard deviation.

The mean was calculated as shown in Equation (2):

(2) $$\bar{X}=\frac{1}{n}\sum_{i=1}^{n}X_{i}$$

where $\bar{X}$ is the sample mean (N), n is the sample size (n = 10 in this experiment), and $X_{i}$ is the measured compressive strength of the i-th pellet (N).

The standard deviation was calculated as shown in Equation (3):

(3) $$\;S=\sqrt{\frac{1}{n-1}\sum_{i=1}^{n}(X_{i}-\bar{X}{)}^{2}}$$

where S is the sample standard deviation (N).

#### 2.3.3. Experiment 3: Optimization of Thermal Parameters

Based on the preliminary results of Experiments 1 and 2, a systematic optimization study of the thermal regime parameters was further conducted. This set of experiments investigated the effects of the following three key thermal parameters on the compressive strength of finished pellets:

- Preheating temperature optimization: The roasting temperature was fixed at 1250 °C, the roasting time was fixed at 25 min, and furnace cooling was used. Under these conditions, the effect of different preheating temperatures (500 °C, 600 °C, 700 °C, 800 °C, and 900 °C) on pellet strength was examined.
- Roasting temperature optimization: The optimal preheating temperature was used as the basis. The roasting time was fixed at 25 min, and furnace cooling was used. Under these conditions, the effect of different roasting temperatures (1100 °C, 1150 °C, 1200 °C, 1250 °C, and 1300 °C) on pellet strength was assessed.
- Cooling regime optimization: The optimal preheating and roasting temperatures were used as the basis. Four cooling methods were compared: water cooling, air cooling, furnace cooling, and cooling after holding at 800 °C. Their effects on finished-pellet compressive strength and internal thermal stress distribution were analyzed.

## 3. Results

### 3.1. Thermal Cracking Behavior Under Varied Heating and Bentonite Content

**Figure 4:**
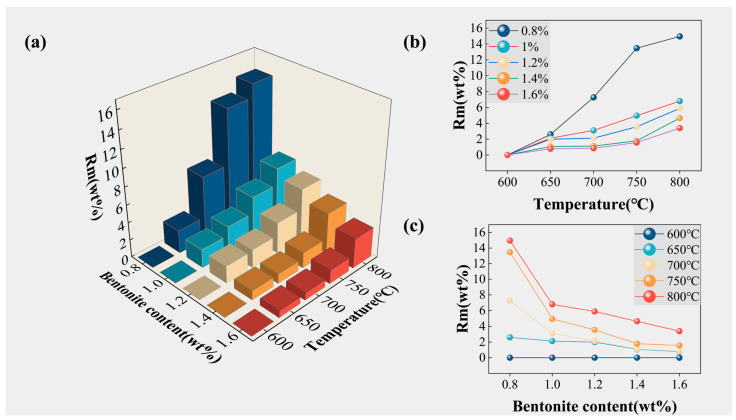
Relationship between preheating temperature, bentonite content, and thermal cracking mass ratio: (**a**) 3D bar chart; (**b**) target preheating temperature vs. thermal cracking mass ratio; (**c**) bentonite addition vs. thermal cracking mass ratio.

Figure 4 shows the thermal cracking mass ratio, *R_m_*, of pellets heated to target preheating temperatures from 600 to 800 °C under a fixed heating duration. Figure 4a,b show that *R_m_* increases markedly as the target temperature rises. This trend is consistent across all bentonite addition levels. The highest *R_m_* values occur at 800 °C. Figure 4a,c further show that *R_m_* decreases nonlinearly as bentonite content increases from 0.8 wt% to 1.6 wt%. After the addition exceeds 1.2 wt%, the reduction rate of *R_m_* becomes less pronounced.

### 3.2. Compressive Strength of Finished Pellets

**Figure 5:**
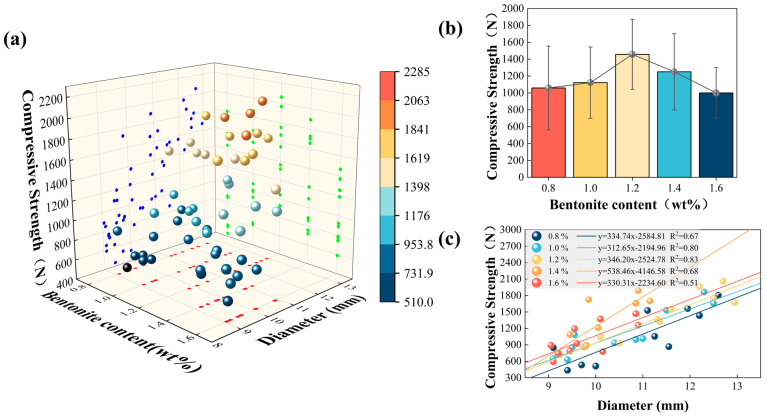
Effect of bentonite addition and pellet diameter on compressive strength: (**a**) 3D scatter plot; (**b**) bentonite addition vs. average compressive strength; (**c**) pellet diameter vs. compressive strength.

Under the fixed roasting conditions, pellets were preheated at 700 °C for 10 min and roasted at 1250 °C for 25 min. Figure 5 shows that the compressive strength depends on both bentonite content and pellet diameter. The average strength first increased with increasing bentonite addition and reached a maximum of 1456 ± 604 N at 1.2 wt% bentonite. Further increasing the bentonite content reduced the strength, as shown in Figure 5b. Larger pellets within the 9–13 mm range showed higher average strength than smaller pellets under the same thermal conditions, as shown in Figure 5a,c.

### 3.3. Influence of Thermal Parameters on Pellet Strength

#### 3.3.1. Preheating Temperature

**Figure 6:**
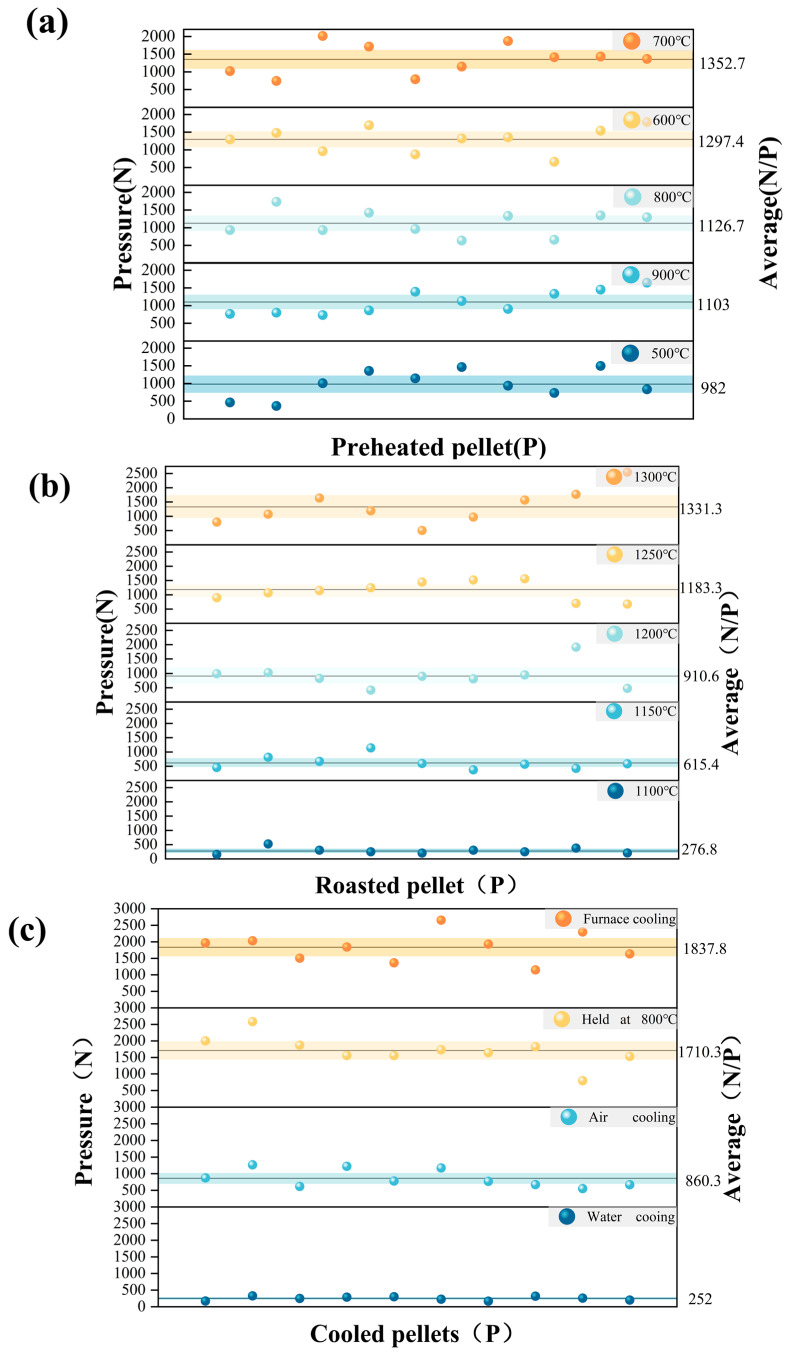
Comparison of pellet compressive strength under different thermal parameters: (**a**) different preheating temperatures; (**b**) different roasting temperatures; (**c**) different cooling methods.

Figure 6a shows the effect of preheating temperature on compressive strength. The strength increased from 982.0 N at 500 °C to a maximum of 1352.7 N at 700 °C. When the preheating temperature was further increased, the strength decreased to 1126.7 N at 800 °C and 1103.0 N at 900 °C. Therefore, 700 °C was selected as the optimal preheating temperature.

#### 3.3.2. Roasting Temperature

Figure 6b shows the effect of roasting temperature on compressive strength at a fixed preheating temperature of 700 °C and a roasting time of 25 min. The strength increased progressively from 1100 °C to 1300 °C. It reached 1183.3 N at 1250 °C and 1331.3 N at 1300 °C. Considering the limited strength improvement and the additional energy consumption at 1300 °C, 1250 °C was selected as the recommended roasting temperature.

#### 3.3.3. Cooling Method

Figure 6c compares the compressive strength under different cooling regimes. Furnace cooling produced the highest average strength of 1837.8 N, followed by air cooling. In contrast, water quenching caused severe strength degradation and nearly complete pellet disintegration. These results indicate that slow cooling is essential for reducing internal thermal stress and maintaining pellet integrity.

## 4. Discussion

### 4.1. Microscopic Mechanism of Thermal Cracking

The intensification of thermal cracking with rising preheating temperature is fundamentally attributable to the temperature sensitivity of goethite dehydroxylation kinetics. Near 800 °C, hydroxyl groups are removed rapidly. Water vapor is generated faster than it can diffuse through the pore network. As a result, internal pressure exceeds the fracture strength of the nascent hematite skeleton. Bentonite mitigates this effect through physical pore modification. Montmorillonite platelets fill the intrinsic micropores. They reconstruct isolated and tortuous pores into an interconnected capillary network, which enables smooth vapor escape and avoids localized stress concentration. Beyond 1.2 wt% bentonite, the pore network connectivity approaches saturation, and further improvement becomes limited. The relatively high data dispersion of *R_m_* in the high-temperature range reflects the inherent pore structure inhomogeneity of Guisha limonite particles.

### 4.2. Microscopic Mechanism of Mechanical Performance

The non-monotonic trend of compressive strength with increasing bentonite content reflects the dual role of the binder during roasting. At moderate additions, montmorillonite reacts with SiO_2_ and alkaline gangue components at approximately 1100–1200 °C, generating a low-melting-point silicate liquid phase that spreads along hematite grain boundaries. This liquid-phase-assisted sintering accelerates grain boundary diffusion and promotes crystalline bridge formation between grains, providing the microstructural basis for the peak strength at 1.2 wt% bentonite. Excessive bentonite, however, produces a continuous brittle glassy phase upon cooling, which weakens intergranular bonding and introduces residual tensile stress from thermal expansion mismatch with the hematite matrix. Regarding pellet diameter, larger pellets (9–13 mm) exhibit higher strength due to their smaller specific surface area, which enables more gradual heating and uniform gas escape, leading to denser consolidation. The relatively large standard deviation (±604 N, ~41% of the mean) reflects inherent microstructural inhomogeneity—uneven mineral distribution, local mixing variations, and inconsistent local consolidation—rather than measurement error.

It should be noted that the proposed liquid-phase-assisted bonding mechanism is inferred from the observed strength evolution and the known high-temperature behavior of bentonite-containing iron ore pellets. Further SEM-EDS or phase analysis of roasted pellets would be useful for directly verifying the distribution of the silicate phase and the morphology of intergranular bonding.

### 4.3. Microscopic Basis for Thermal Regime Optimization

The strength trend in the preheating temperature tests reflects the kinetic balance of goethite dehydroxylation. At 700 °C, the highest strength (mean 1352.7 N) is achieved because the dehydroxylation rate matches the vapor diffusion rate through the pore network. This allows complete hydroxyl removal without localized pressure buildup. Below 700 °C, incomplete dehydroxylation leaves residual hydroxyl groups in the hematite lattice. These groups cause secondary cracking during subsequent roasting, resulting in lower strength at 500–600 °C. Above 700 °C, dehydroxylation becomes excessively rapid. The resulting internal stress exceeds the strength of newly formed interparticle bonds before sufficient consolidation can occur. This leads to microcracks and strength reduction at 800–900 °C.

The progressive strength increase with roasting temperature from 1100 °C to 1300 °C is attributed to the activation of solid-state diffusion mechanisms. Higher temperatures provide a greater driving force for the migration of O^2−^ and Fe^3+^ ions between adjacent hematite grains, promoting the growth and interconnection of crystalline bridges. The diminishing strength gain from 1250 °C to 1300 °C suggests that the diffusion-controlled consolidation approaches completion, making 1250 °C the recommended temperature when energy economy is considered.

The dramatic differences among cooling methods arise from thermal stress relaxation. During furnace cooling, the slow temperature decline minimizes thermal gradients within each pellet, allowing stresses to dissipate through atomic diffusion and grain boundary sliding, resulting in the highest strength (1837.8 N). Water quenching generates extreme surface tensile stress as the exterior contracts rapidly while the interior remains hot and expanded, exceeding the bonding strength of newly formed grain boundaries and causing near-complete disintegration (252 N). Air cooling produces intermediate results as the cooling rate falls between these two extremes.

### 4.4. Microstructural Basis and Quantitative Optimization of the Process Trade-Off

The above micro-mechanism analysis provides a structural explanation for the difference between the 1.2 wt% and 1.4 wt% bentonite formulations. Under the 1.2 wt% condition, a moderate amount of liquid phase promotes sufficient interconnection among hematite grains. This condition has low glass phase content and high grain boundary bonding strength, thereby achieving optimal compressive strength. Under the 1.4 wt% condition, the thermal cracking suppression gained from the increased liquid phase outweighs the strength loss. The connected pore network allows for more gradual dehydration. Meanwhile, the decrease in strength remains within an acceptable range.

To elevate the above qualitative trade-off to a quantitative decision, this study adopts the Min–Max normalization method to construct a comprehensive performance evaluation function. The normalization procedure is as follows:

For compressive strength, the finished pellets under fixed roasting conditions (preheating at 700 °C for 10 min, roasting at 1250 °C for 25 min) are analyzed. The normalization formula is shown in Equation (4):

(4) $$\sigma_{i}=\frac{S_{i}-S_{min}}{S_{max}-S_{min}}$$

Here, $\sigma_{i}$ is the normalized compressive strength. The average compressive strength of the i-th bentonite group is expressed in N. The maximum compressive strength among all groups is 1456 N, which appears in the 1.2 wt% group. The minimum value is 870 N, which appears in the 0.8 wt% group. $\sigma_{i}$ ranges from 0 to 1. A larger value indicates better mechanical performance.

For anti-decrepitation ability (characterized by $R_{m}$, a negative indicator processed through positive transformation), the normalization formula is shown in Equation (5):

(5) $$\rho_{i}=1-\frac{R_{m,max}-R_{m,i}}{R_{m,max}-R_{m,min}}$$

where $\rho_{i}$ denotes the normalized anti-decrepitation ability. $R_{m,i}$ is the thermal cracking mass ratio of the *i*-th bentonite group at a preheating temperature of 700 °C (%). $R_{m,max}$ and $R_{m,min}$ represent the maximum and minimum $R_{m}$ values among all groups, corresponding to the 0.8 wt% and 1.6 wt% bentonite groups, respectively. The value of $\rho_{i}$ ranges from 0 to 1, and a larger value indicates stronger anti-decrepitation ability.

On the basis of the above normalization, a weighted composite score function is constructed as shown in Equation (6):

(6) $$Z_{i}=w_{1}\cdot \sigma_{i}+w_{2}\cdot \rho_{i}$$

where $w_{1}$ and $w_{2}$ are the weighting coefficients for compressive strength and anti-decrepitation ability, respectively, with $w_{1}$ + $w_{2}$ = 1. By adjusting the weights, different industrial decision-making scenarios can be simulated.

**Figure 7:**
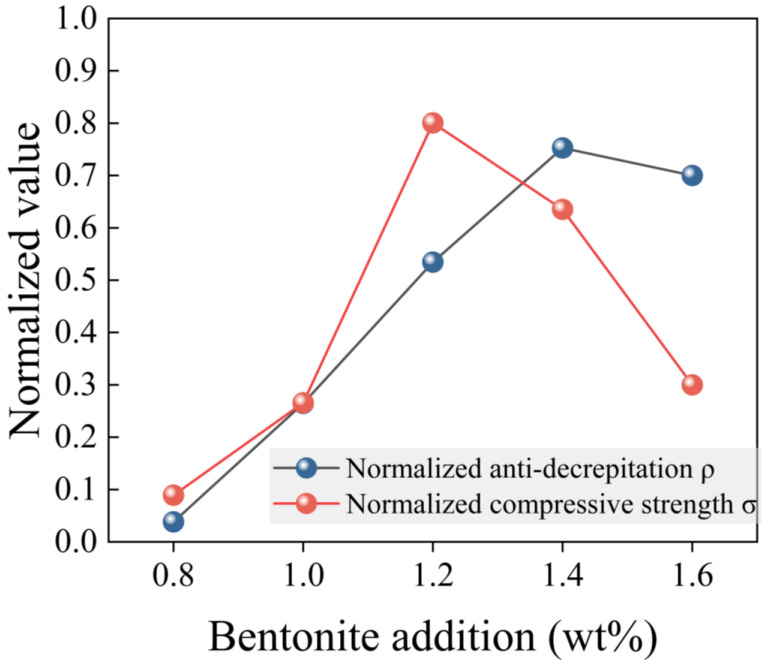
Comparison of normalized indicators and weighted composite scores for different bentonite addition levels under two decision-making modes.

The two weighting modes were designed to represent two typical industrial scenarios, and the calculated composite scores for different bentonite addition levels are shown in Figure 7. In the strength-prioritized mode, compressive strength was assigned a larger weight ($w_{1}$ = 0.7), whereas anti-decrepitation ability was assigned a smaller weight ($w_{2}$ = 0.3). This mode reflects the operating condition in which mechanical integrity is the dominant requirement for burden support. The 1.2 wt% bentonite group achieved the highest composite score under this mode, confirming its suitability when mechanical performance is prioritized. In the safety-prioritized mode, anti-decrepitation ability was assigned a larger weight ($w_{2}$ = 0.7), whereas compressive strength was assigned a smaller weight ($w_{1}$ = 0.3). This mode represents conditions in which suppressing pulverization is more critical for maintaining blast furnace permeability under unstable raw ore conditions. In this case, the 1.4 wt% bentonite group ranked first, indicating that a moderate increase in bentonite dosage can improve the safety margin against decrepitation, although it is accompanied by a certain loss in compressive strength.

These two operational windows are not mutually exclusive. Instead, they provide operational flexibility. The choice between them depends on the specific requirements of the downstream ironmaking process and the intrinsic variability of the raw ore feed. In industrial practice, the inherent discreteness of pellet strength must be fully considered. The standard deviation of ±604 N in this experiment indicates that individual pellet strength may fluctuate over a relatively wide range even under the same optimal formulation. Therefore, an appropriate fluctuation margin should be reserved based on the mean strength during blast furnace operation. This helps ensure the reliability and gas permeability of the overall burden structure.

## 5. Conclusions

This study investigated the thermal decrepitation and consolidation behavior of Guisha limonite pellets through a three-factor experimental design involving bentonite ratio, preheating regime, and pellet size. The main conclusions are as follows:

- Multi-factor synergistic regulation mechanism of thermal cracking behavior: Under simulated thermal shock conditions with a fixed heating time, higher preheating temperatures lead to more vigorous goethite dehydroxylation. This causes the thermal cracking mass ratio to rise significantly. Increasing the bentonite addition effectively suppresses thermal cracking. The underlying mechanism involves montmorillonite filling the intrinsic micropores of the ore and optimizing water-vapor escape channels. This transforms dehydration from an “explosive release” mode to a “gradual diffusion” mode. Compared with existing single-factor studies, this work reveals, for the first time, the interactive influence of bentonite ratio and heating rate on thermal cracking behavior. It provides a scientific basis for multi-variable synergistic regulation in pellet preheating regime design.
- Adapted optimized thermal parameters: Based on the mineralogical composition and high loss-on-ignition characteristics of Guisha limonite, the optimal thermal regime is determined as a preheating temperature of 700 °C, a roasting temperature of 1250 °C, and strict implementation of slow furnace cooling. Slow furnace cooling avoids internal stress damage caused by rapid cooling. This set of parameters can serve directly as a benchmark process window for pellet production of similar high-crystal-water iron ores. It helps resolve the problems of insufficient strength and pulverization caused by thermal decrepitation of this type of ore.
- Process balancing strategy based on Min–Max normalization and weighted scoring: The compressive strength and thermal decrepitation risk of pellets were comprehensively weighed. A Min–Max normalization and weighted scoring method was adopted. On this basis, a differentiated process decision-making scheme was proposed for the first time. When mechanical performance is the primary objective, 1.2 wt% bentonite is recommended. When suppressing thermal cracking and ensuring smooth blast furnace operation are the primary safety premises, 1.4 wt% bentonite is recommended. This dual-track strategy elevates limonite pellet production from traditional “empirical trial-and-error” to “quantitative decision-making.” It provides an actionable basis for industrial sites to flexibly switch process parameters according to blast furnace conditions and raw material fluctuations.

From an industrial application perspective, the optimized thermal regime of preheating at 700 °C, roasting at 1250 °C, and slow furnace cooling can be aligned with the temperature-zone design of the grate-kiln process. The proposed dual-window strategy provides operators with a flexible basis for selecting 1.2 wt% bentonite when strength is prioritized or 1.4 wt% bentonite when anti-decrepitation safety is prioritized. This approach can help reduce pellet quality fluctuations, improve burden permeability, and enhance the stability of blast furnace operation.

## Figures and Tables

**Table 1:** Main chemical composition of Guisha limonite (wt%) [28].

TFe	FeO	SiO_2_	Zn	Na_2_O	TiO_2_	V_2_O_5_	S	K_2_O	MnO	Cu	Pb	LOI
54.67	0.29	4.04	0.018	0.001	0.27	0.04	0.048	0.095	3.47	0.009	0.009	14.82

**Table 2:** Physicochemical performance indices of the bentonite used.

Moisture (%)	Colloid Index (%/3 g)	Swelling Capacity (mL g^−1^)	Water Absorption (%)	Methylene Blue Index (g/100 g)	Montmorillonite Content (%)
9.38	20.0	34.5	408	38.0	85.97

## Data Availability

The original contributions presented in this study are included in the article. Further inquiries can be directed to the corresponding author.
